# Normal Hematopoietic Stem Cell Function in Mice with Enforced Expression of the Hippo Signaling Effector YAP1

**DOI:** 10.1371/journal.pone.0032013

**Published:** 2012-02-21

**Authors:** Lina Jansson, Jonas Larsson

**Affiliations:** Molecular Medicine and Gene Therapy, Lund Stem Cell Center, Lund University, Lund, Sweden; Emory University, United States of America

## Abstract

The Hippo pathway has recently been implicated in the regulation of organ size and stem cells in multiple tissues. The transcriptional cofactor yes-associated protein 1 (Yap1) is the most downstream effector of Hippo signaling and is functionally repressed by the upstream components of the pathway. Overexpression of YAP1 stimulates proliferation of stem and progenitor cells in many tissues, consistent with inhibition of Hippo signaling. To study the role of Hippo signaling in hematopoietic stem cells (HSCs), we created a transgenic model with inducible YAP1 expression exclusively within the hematopoietic system. Following 3 months induction, examination of blood and bone marrow in the induced mice revealed no changes in the distribution of the hematopoietic lineages compared to control mice. Moreover, the progenitor cell compartment was unaltered as determined by colony forming assays and immunophenotyping. To address whether YAP1 affects the quantity and function of HSCs we performed competitive transplantation experiments. We show that ectopic YAP1 expression does not influence HSC function neither during steady state nor in situations of hematopoietic stress. This is in sharp contrast to effects seen on stem- and progenitor cells in other organs and suggests highly tissue specific functions of the Hippo pathway in regulation of stem cells.

## Introduction

The first components of the Hippo tumor suppressor pathway were discovered two decades ago in *Drosophila* mosaics studies as regulators of cell shape and cell proliferation [1], [2]. Subsequently, all the major cytosolic components have been established and the pathway is now understood to be organized as a kinase-signaling cascade that negatively regulates the downstream effector Yorkie [3]. The function of the pathway is largely evolutionary conserved and mammalian homologs corresponding to all *Drosophila* Hippo proteins have been identified [4], [5], [6].

As homologs of *Drosophila* Hippo (Hpo), the mammalian STE20-like protein kinase 1 and 2 (Mst1/2) make up one of the two core kinase groups in the Hippo pathway. Mst is stabilized by interacting with the Salvador homolog 1 (Sav1) and activates the downstream large tumor suppressor homolog 1 and 2 (Lats 1/Lats 2) via phosporylation [7]. Lats 1/2 interact with Mps One Binder kinase activator-like (Mob1), homolog of the *Drosophila* mats, and then in turn phosphorylate the Yorkie homolog Yes associated protein 1 (Yap1). Phosphorylated Yap1 contains a binding site for 14-3-3 proteins, which promotes cytosolic retention and prevents Yap from translocating to the nucleus [5], [8]. Both the *Drosophila* Yorkie and its mammalian homolog Yap1, contain highly conserved WW-domains. These domains recognize proline-rich motifs facilitating protein–protein interactions [9]. In the nucleus, Yap1 functions as a transcriptional coactivator, initiating transcription in complex with various transcription factors, such as p73, EGR-1, Runx 1/2 and particularly the TEA domain (TEAD) family [10], [11]. The interactions with TEAD transcription factors are the only known interactions conserved from *Drosophila* to mammals [12]. A main biological function of Yap1 is to promote cell proliferation through regulation of cell cycling and apoptosis. These functions are thus counteracted by the upstream Hippo components, resulting in a tight regulation of tissue homeostasis, as demonstrated in mouse models of altered Hippo signaling. Zhou and colleagues established that combined Mst1/Mst2 deficiency in the liver results in massive overgrowth and hepatocellular carcinoma as the loss of Mst1/Mst2 signaling abrogates Yap1 phosphorylation, leading to enhanced Yap1 activity in the nucleus and an increased transcriptional activity. Consistent with these consequences of perturbed Hippo signaling, several studies have demonstrated that overexpression of YAP1 in the liver results in a dramatic increase in cell proliferation and organ size [4], [13].

The profound role of Hippo signaling in regulating tissue homeostasis across different species raises the possibility of a functional importance in stem cells. In a transcriptional profiling study by Ramalho-Santos et al, comparing embryonic, neural and hematopoietic stem cells showed that Yap1 was one of a few genes with a consistently higher expression across the stem cell fractions compared to differentiated cells [14]. More recently, these observations have been substantiated through functional studies of Yap1 in various stem cell types where Yap1 has been established as a vital factor in stem cell maintenance and proliferation. Cao and colleagues showed that YAP1 regulates neural progenitor cell number in the chick neural tube [15]. It was further demonstrated that Yap1 is necessary for maintained pluripotency in murine embryonic stem (ES) cells and that ectopic expression of YAP1 prevents ES cell differentiation [16], [17]. Finally, overexpression of YAP1 in the mouse intestine leads to expansion of multipotent progenitors [13]. Taken together, this provides evidence that YAP1 functions as a stem cell regulator.

While the Hippo pathway and Yap1 has been extensively studied in other tissues, its function in the hematopoietic system remains largely unknown. Here we study the role of Hippo signaling in hematopoiesis by conditionally overexpressing YAP1 within the hematopoietic system in vivo.

## Methods

### Ethics statement

This study was carried out in strict accordance with the recommendations in the Guide for the Care and Use of Laboratory Animals of the National Institutes of Health. All animal experiments were reviewed and approved by the Lund/Malmö Local Ethical committee as defined in the ethical permit with number M217-09.

### Tet-YAP vector construction

The tet-YAP expression vectors was constructed using wildtype human YAP1 cDNA (5747370, Open Biosystems) and YAP1 DNA synthesized with a Serine 127 to Alanine mutation (ATG:biosynthetics GmbH, Merzhausen, Germany). Both were inserted into the pBS31 targeting vector using EcoRI and MluI restriction sites [18].

### ES cell culture and gene targeting

Embryonic stem cell line KH2 [18] was cultured on irradiated murine embryonic fibroblast (MEF) cells according to standard procedure. Briefly, cells were seeded at a density of 3×10^4^ cells/cm^2^ in DMEM supplemented with LIF at 1000 units/ml (ESGRO®, Millipore), 15% ES qualified fetal bovine serum, non essential amino acids and sodium pyruvate (GIBCO), and passaged every other day. Gene targeting was performed as follows; tet-YAP vectors were co-electroporated with a Flp recombinase-expressing vector, pCAAGS-Flp, and seeded on MEFs. Colonies were picked following 7 days of hygromycin selection and screened for integration of the vector into the ColA1 locus with PCR primers: tccctcacttctcatccagatatt, agtcttggatactccgtgaccata, ggacaggataagtatgacatcatcaa. YAP1 overexpression was induced with doxycycline at a concentration of 1 µg/ml during 3 days.

### Generation of tet-YAP1- fetal liver cells and YAP1 chimeric mice

A correctly targeted ES clone was injected into W^41^/W^41^ blastocysts (CD45.2, The Jackson Laboratory, Bar Harbor, USA) and transferred to pseudo pregnant foster mothers. Fetal liver cells were isolated from day 14.5 embryos as described before [19]. Lethally irradiated (900 cGy) C57bl/6/SJL mice (CD45.1, in-house breeding) were then injected intravenously with 2×10^6^ unfractionated fetal liver cells to generate primary tet-YAP1 chimeric mice. Reconstitution was confirmed six weeks post-transplant where after mice were induced during 12 or 15 weeks (wildtype and mutant YAP1 respectively) by supplementing the drinking water with of 2 mg/ml doxycycline and 10 mg/ml sucrose. Secondary recipients were transplanted with 2×10^6^ bone marrow cells from primary mice and an identical number of competitor cells from congenic C57Bl/6×SJL (CD45.1/CD45.2, in-house breeding) mice.

### Generation of YAP^mut^ germline mice

The verified tet-YAP^mut^ ES clone was injected into W41/W41 blastocysts, which were transferred into pseudo pregnant foster mothers and carried to terms. Male mice from the litters were crossed with C57/bl6 mice and progeny genotyped for the tet-YAP^mut^ modification at the Cola1a locus (PCR primers same as before) and the rtTa modification at the ROSA26 locus with PCR primers: aaagtcgctctgagttgttat, gcgaagagtttgtcctcaacc, ggagcgggagaaatggatatg. Germline progeny was then further intercrossed to produce mice with different combination of genotypes.

### Real-time PCR

Whole bone marrow was isolated form C57BL/6 mice (Taconic) and enriched for c-kit positive cells using CD117 MicroBeads and MACS separation columns (Miltenyi Biotec). Cells were stained with antibodies against CD34, Flt3, Sca-1, c-kit and lineage markers CD3, CD4, CD8, Gr1, Mac-1, B220 and Ter119. All antibodies from Biolegend. Long-term HSCs (Lin-, Sca-1+, c-kit+(LSK), Flt3−, CD34−), short-term HSCs (Lin−, Sca-1+, c-kit+, Flt3−, CD34+), lymphoid myeloid primed progenitors (Lin−, Sca-1+, c-kit+, Flt3+, CD34+) and lineage positive (Lin+) cells were sorted using the FACS Aria (Becton Dickinson). RNA was isolated using the Mini RNAeasy kit (Qiagen) and cDNA transcribed with SuperScript III reverse trancriptase (Invitrogen). Real-time PCR was performed with the Taqman Assay System and primer sets from Applied Biosciences.

### Flow cytometry

Intracellular levels of YAP expression were measured with flow cytometry using primary anti-YAP antibody from Cell Signaling (#4912) and secondary anti-rabbit donkey Ig DyLight649 antibody (Biolegend). Cells were fixed with 4% paraformaldehyde (Sigma) and permeabilized in 0,5% Saponin (Sigma).

Additional antibodies used for determining lineage distribution and HSC immunophenotype were anti-CD45.1 and CD45.2 (Biolegend).

### Colony forming assay

Whole bone marrow cell suspension was plated in methylcellulose medium (M3434, Stem Cell Technologies) and scored for colony formation according to the manufacturer's instruction. Doxycycline was added to the medium at a concentration of 1 µg/ml.

### Western blot

ES cells were resuspended in Laemli buffer (BioRad) according to manufacturer's instructions, denatured by heating at 95°C for 10 min and then centrifuged at 4°C. The supernatant was loaded on NuPage® Gels and blotted using the iBlot® Dry Blotting system (Invitrogen). Membranes were probed with primary antibody against YAP1 (same as before) and α-tubulin (#7291,Abcam). ECL™-HRP linked secondary antibody and Amersham™ ECL Plus detection kit were both from GE Healthcare. Fractionation of nuclear fraction was performed as described before [20].

## Results

### Components of the Hippo signaling pathway are expressed in HSPCs

**Figure 1:**
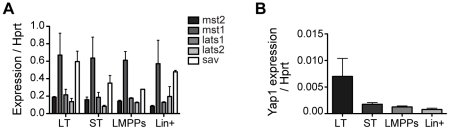
Components of the Hippo signaling pathway are expressed in hematopoietic stem and progenitor cells. mRNA expression levels of upstreams components of the Hippo pathway (**A**) and the downstream effector Yap1 (**B**) in different stem and progenitor compartments. Values are normalized against Hprt1 for n = 2 (**A**) and n = 3 (**B**). Error bars represent SEM. Abbreviations: LT, long-term HSCs; ST, short-term HSCs; LMPPs, lymphoid myeloid primed progenitor; Lin+, lineage positive.

Recent work has shown that Hippo signaling through Yap1 regulates a variety of tissue stem cells, as well as ES cells, by modulating cell proliferation and apoptosis [15], [17]. To assess the role of Yap1 in hematopoietic cells we first evaluated the presence of components of the Hippo signaling pathway in different subsets of bone marrow hematopoietic cells. We used RT-qPCR to determine mRNA levels of the core components Lats1/2, Mst1/2, Sav1 as well as Yap1 in long-term (LT) repopulating HSC, short-term (ST) repopulating HSCs, lymphoid myeloid primed progenitors (LMPP) and mature, lineage positive cells. The upstream kinases Lats1/2 and Mst1/2 as well as the WW domain Sav1 were clearly detected across all these subsets (Figure 1A) in contrast to Yap1 which was detected at low levels in LT-HSCs and was barely detectable in the other subsets (Figure 1B). Thus, while all components upstream of Yap1 show widespread expression in hematopoietic cells, Yap1 expression is more restricted to the primitive HSC fraction.

### Generation of a model for inducible expression of YAP1 in the hematopoietic system

**Figure 2:**
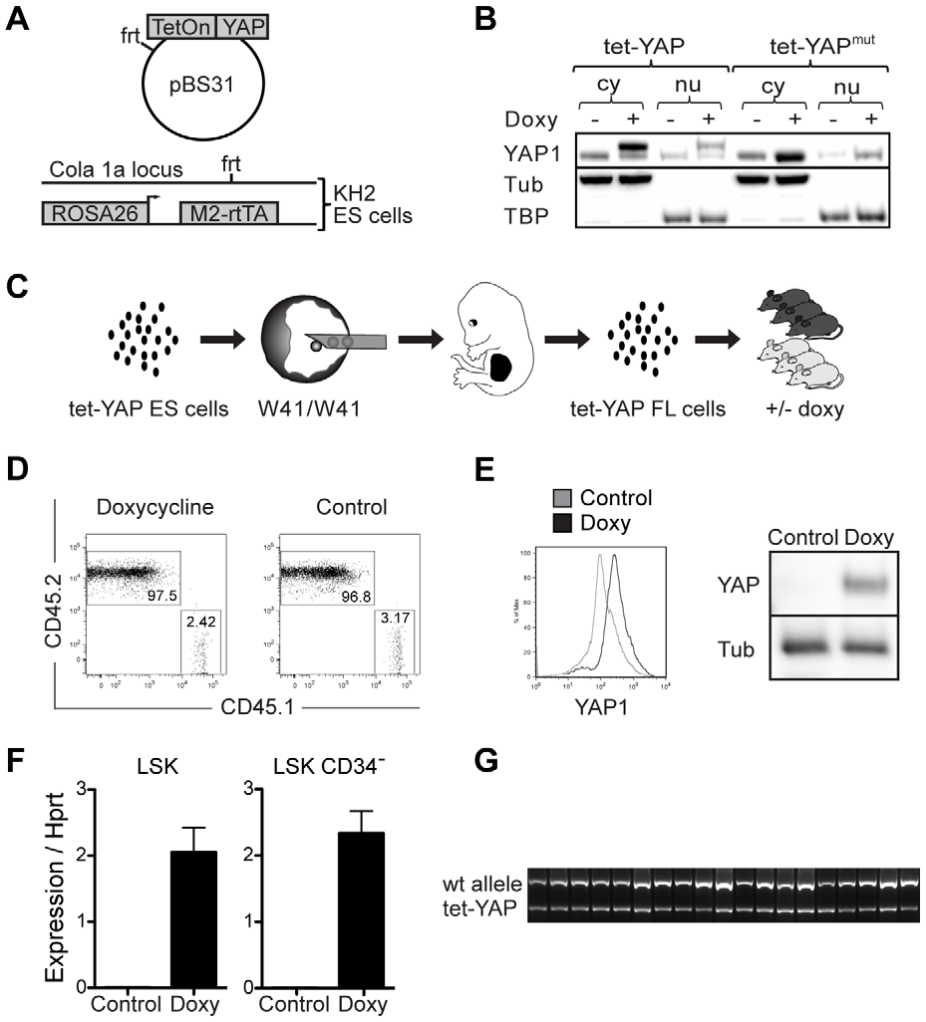
Generation of a mouse model with inducible YAP1 overexpression in the hematopoietic system. (**A**) ES cell targeting vector containing tetracycline inducible promoter driving hYAP cDNA expression. The vector was targeted to the Cola1a locus of the KH2 ES cell line using flp/FRT recombination. The endogenous Rosa26 promoter drives expression of the M2rtTA transactivator. (**B**) Western blot depicting overexpression of YAP1 in ES clones with wildtype (tet-YAP) or mutated constitutively active (tet-YAP^mut^) cDNA. Cells were cultured with (+) or without (−) doxycycline for 5 days and then harvested and separated into cytosolic (cy) and nuclear (nu) fractions. The membrane was probed with antibodies against YAP1, Tata-binding-protein (TBP), and alpha-tubulin (α-Tub). The wildtype human YAP1 protein is a longer isoform and is represented by the higher molecular weight band whereas the lower band represents the murine and mutant human protein. (**C**) An overview of the experimental setup. Briefly, ES cells (CD45.2) were targeted with a tetracycline inducible YAP1 cDNA vector, injected into E3.5 blastocysts from W^41^/W^41^ mice and then transferred to a foster mother. Fetal liver (FL) cells were isolated from E14.5 embryos and whole cell suspension was transplanted into irradiated recipients (CD45.1), generating tet-YAP1 hematopoietic chimeras. (**D**) Representative example of reconstitution levels in control and doxycycline treated tet-YAP1 chimeric mice. Bone marrow cells were stained for congenic markers CD45.2 for donor tet-YAP1 cells and CD45.1 for recipient mice. (**E**) Analysis of expression levels of YAP1 in the bone marrow of control and doxycycline treated tet-YAP mice using intracellular flow cytometry (left panel) and Western Blot (right panel). (F) Expression of YAP^mut^ mRNA was verified in sorted progenitor (LSK) and stem cell (LSK CD34^−^) fractions using RT-qPCR. (**G**) PCR confirmation of tet-YAP^mut^ donor chimerism in hematopoietic stem and progenitor cells >30 weeks after transplantation of tet-YAP FL cells. Bone marrow cells were plated in methylcellulose medium and PCR was performed on individual colonies. A minimum of 24 colonies was screened per mouse. Abbreviations: M2-rtTA, reverse tetracycline transactivator; Tet, tetracycline; ES cells, murine embryonic stem cells; FL, fetal liver; LSK, Lineage negative, Sca1 positive, kit positive cells.

We next generated mice with inducible YAP1 overexpression in hematopoietic cells. Targeting constructs containing either wildtype YAP1 cDNA (YAP) or a constitutively active mutant (YAP^mut^, Figure S1A–B) under control of a tet-ON promoter were integrated into the ColA1 locus of KH2 ES cells (Figure 2A) as described before [18], [21]. Doxycycline-inducible expression was confirmed by flow cytometry (Figure S1C) and nuclear localization of YAP1 was verified by Western blot on ES cell extracts (Figure 2B). The wildtype and mutant human isoform were clearly detected in both cytosolic and nuclear fractions when doxycycline was added to the cultures (Figure 2B, Lane 2,4 and 6,8) consistent with a high translation rate in the cytosol and translocation to the nucleus. The wildtype isoform has a higher molecular weight than the endogenous murine protein and the mutant isoform. Next, as outlined in figure 2C, tet-YAP ES cells were injected into blastocysts from the HSC-deficient W^41/41^ strain and transferred to foster mothers. We have previously demonstrated that W^41^/W^41^ blastocyst complementation generates embryos with fetal liver HSCs of practically pure ES cell origin [21]. Fetal liver cells were transplanted into lethally irradiated recipients to generate cohorts of mice with tet-YAP transgenic hematopoiesis (Figure 2C). This model system allows for tightly controlled transgene expression within the hematopoietic system including all HSCs [21]. After six weeks, complete hematopoietic reconstitution from tet-YAP fetal liver cells was confirmed in all mice (Figure 2D). Overexpression of YAP1 in hematopoietic cells was verified in mice treated with doxycycline using flow cytometry and Western blot (Figure 2E). Expression specifically in the stem and progenitor compartments was confirmed with RT-qPCR (Figure 2F). Furthermore, mice that were followed for up to a year showed hematopoiesis of complete transgenic origin (Figure 2G). Both the wildtype and mutant YAP1 constructs showed the expected phenotypes when expression was induced in other tissues (Figure S2) [4], [13].

### Normal hematopoiesis in YAP1 induced mice

**Figure 3:**
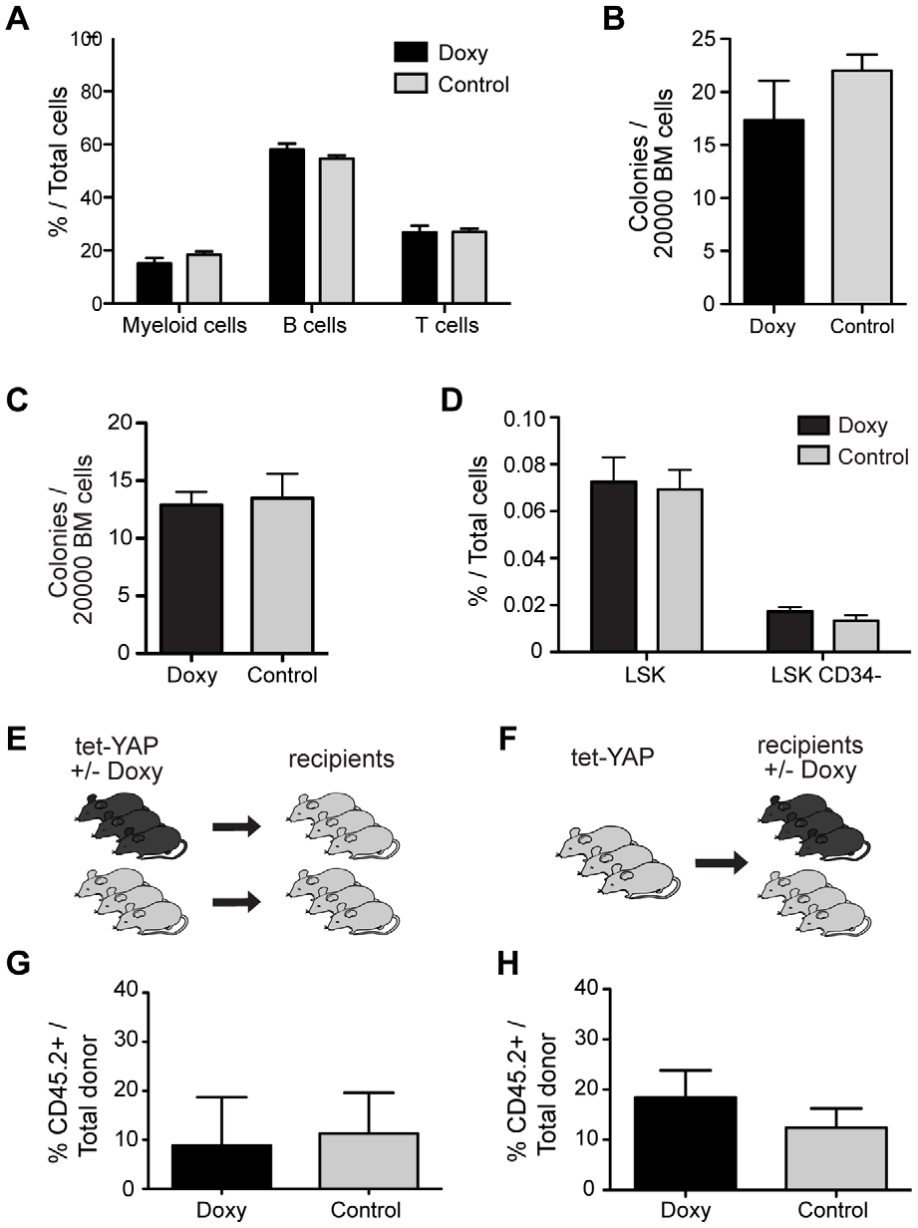
Normal hematopoiesis in YAP1 induced mice. Irradiated mice were transplanted with tet-YAP1 fetal liver cells and analyzed after 12 weeks of doxycycline induction. (**A**) The distribution of different cell lineages in peripheral blood using flow cytometry. Myeloid cells were Mac-1, Gr1 positive; B cells B220 positive and T cells CD3 positive. (**B**) The total number of methylcellulose colonies per 20000 total bone marrow cells from control and doxycycline treated mice. (**C**) The total number of methylcellulose colonies per 20000 total bone marrow cells from untreated tet-YAP1 mice with or without doxycycline in the medium. (**D**) The frequency of hematopoietic stem and progenitor cells in total bone marrow of mice after YAP1 overexpression. (**E**) Functional analysis of the stem cell compartment in tet-YAP mice using competitive transplantations. Total bone marrow cells (CD45.2) from tet-YAP1 reconstituted mice were transplanted into secondary recipients together with an equal number of wildtype competitor cells (CD45.1/45.2) 12 weeks after doxycycline induction. (**F**) Cells from control and doxycycline treated mice were transplanted into irradiated recipients without treatment and in paralell (**G**) control bone marrow cells were transplanted, with competitors, and the recipient mice were administered doxycycline directly upon injection. (**H**) Engraftment levels in peripheral blood 14 weeks after transplantation of induced and un-induced tet-YAP1 cells and (D) engraftment after transplantation of untreated tet-YAP1cells with doxycycline administration upon injection. Error bars represent SEM for n = 7 (A–D, G) and n = 3 (H). Abbreviations: LSK, Lineage negative, Sca1 positive, kit positive cells.

To determine the consequences of enforced YAP1 expression within the hematopoietic system, we analyzed the induced tet-YAP mice after administering doxycycline for 12 weeks. Examination of blood and bone marrow in the induced mice revealed no changes in the distribution of the hematopoietic lineages compared to control mice (Figure 3A). Moreover, no difference in the number of colony forming progenitor cells was found (Figure 3B,C) and the frequency of the LSK multipotent progenitor population and the CD34 negative HSC population was unaltered (Figure 3D). Taken together, the overall hematopoietic profile, based on analysis of cell surface marker expression and progenitor assays was normal in YAP1 induced mice.

### Enforced expression of YAP1 does not alter HSC number and function measured by competitive transplantation

To further investigate if YAP1 expression alters the number or function of hematopoietic stem cells *in vivo* we performed competitive transplantation experiments. Unfractionated bone marrow cells from treated and control tet-YAP mice (expressing the CD45.2 cell surface marker) were injected together with equal numbers of wildtype competitor cells (expressing both CD45.1 and CD45.2) into lethally irradiated CD45.1 recipients to determine if the frequency of functionally defined stem cells was altered after YAP1 expression (Figure 3E). Since there is no induction of YAP1 expression during regeneration in the recipient mice, this setup confines the readout to an assessment of the size of the HSC pool present in the tet-YAP mice before transplantation. Analysis of donor contribution following long-term reconstitution (Figure 3F) showed similar engraftment levels between mice transplanted with cells from induced tet-YAP mice and controls, demonstrating that overexpression of YAP1 had not influenced the HSC pool size during steady-state hematopoiesis. To further test the consequences of YAP1 overexpression on HSC function during hematopoietic regeneration, we performed competitive transplantations with cells from non-induced tet-YAP mice into recipients that were administered doxycycline upon transplantation (Figure 3G). However, we observed no difference in reconstitution capacity between mice with enforced YAP1 expression and controls (Figure 3H). Thus, we conclude that ectopic YAP1 expression does not influence HSC function, neither during steady state, nor in situations of hematopoietic stress.

### Inducible tissue-restricted expression of a constitutive active form of YAP1 does not alter HSC function

**Figure 4:**
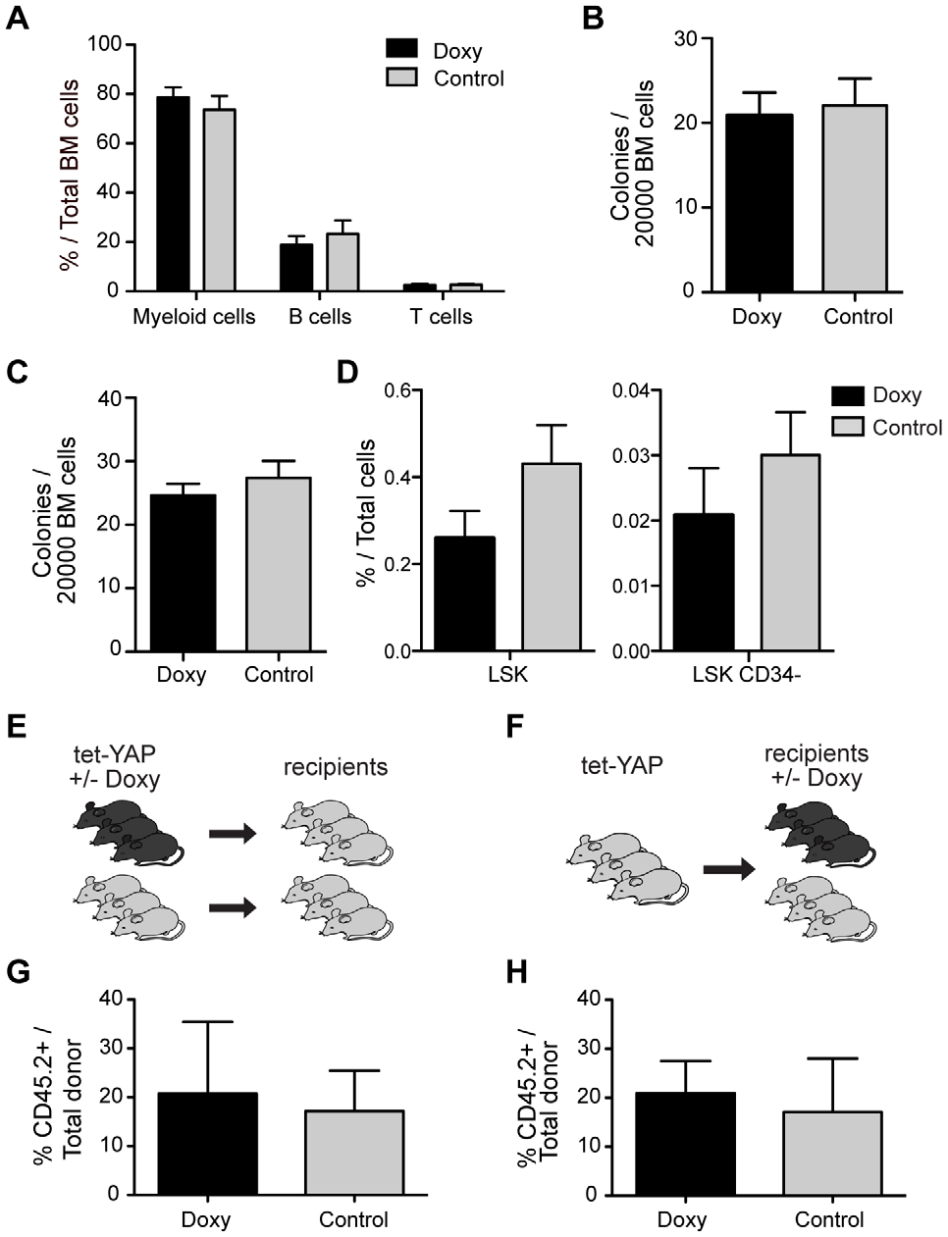
Inducible tissue-restricted expression of a constitutive active form of YAP1 does not alter HSC function. Primary recipients were transplanted with tet-YAP^mut^ fetal liver cells after 15 weeks of induction. (**A**) Lineage distribution in bone marrow of tet-YAP^mut^ mice. Myeloid cells were Mac-1, Gr1 positive; B cells B220 positive and T cells CD3 positive. (**B**) Colony formation efficiency of tet- YAP^mut^ bone marrow cells (**C**) Colony formation from tet-YAP^mut^ cells with doxycycline added to the methylcellulose medium. (**D**) Immunophenotyping of progenitor and stem cell compartement using flow cytometric analysis of primary mice (**E**). For the functional analysis of hematopoietic stem cell quantity cells from control and doxycycline treated mice were transplanted into irradiated recipients without treatment. (**F**) In parallel, to evaluate hematopoietic stem cell regeneration, control bone marrow cells were transplanted with competitors, and the recipient mice were administered doxycycline directly upon injection. (G) Reconstitution of tet-YAP1^mut^ cells, treated and untreated, in peripheral blood after 30 weeks. (**G**) Percent tet-YAP^mut^ cells in the bone marrow of recipients 30 weeks after transplantation with induction upon transplantation. Error bars represent SEM for n = 5 (**A**–**F**), **n = 3–4** (**G**) **and n = 5** (**H**). Abbreviations: LSK, Lineage negative-Sca1 positive-kit positive cells.

Since we did not observe a hematopoietic phenotype when expressing wildtype YAP1, we thought it pertinent to investigate the effect of a constitutively active version of YAP1 (YAP^mut^) with an abolished phosphorylation site [5]. It has been shown that YAP1 activity is regulated by phosphorylation of a serine residue (S127), which targets YAP1 for cytoplasmic sequestration and degradation [22]. Using the same approach as for wildtype YAP1 we generated mice, with inducible expression of YAP1^mut^ within the hematopoietic system, and administered doxycycline for 15 weeks. Employing equivalent analysis as for wildtype YAP1, we found no significant difference in lineage distribution, colony formation or number of progenitor cells in primary YAP1^mut^ mice (Figure 4A–D). Furthermore, competitive transplantation experiments showed that YAP1^mut^ had no effect on the quantity or function of stem cells (Figure 4E–G).

Thus, in sharp contrast to what has been described for many other tissue stem cells, increased YAP1 activity does not alter the function of hematopoietic stem and progenitor cells.

## Discussion

An increasing body of work, generated during recent years, implicates Hippo signaling as a key determinant of stem cell function and organ size in a variety of tissues [13], [15], [17]. This has to a large extent been demonstrated in gain-of-function models for YAP1, counteracting upstream Hippo signaling. To clarify whether Hippo signaling plays a significant role in hematopoiesis and in regulation of HSCs we have studied a mouse model for inducible expression of YAP1 within the hematopoietic system. We demonstrate here that enforced expression of neither wildtype nor constitutively active YAP1 alters hematopoiesis or HSC function in vivo. This is in sharp contrast to the effect seen on stem- and progenitor cells in other organs such as the intestine, the brain and the skin, and suggests highly tissue specific functions of the Hippo pathway in regulating stem cells. Thus, while Yap1 has been implicated as a stemness gene in several tissues, its functions are clearly context dependent.

The Hippo pathway is connected to a number of different upstream regulatory elements, most of which remain poorly defined in mammalian cells. Cell-cell interactions that control cell proliferation through contact-mediated inhibition has been attributed to Hippo signaling [23]. However, a distinct property of the hematopoietic system, distinguishing it from solid organs, is the liquid nature of the tissue wherein the cells reside; blood and bone marrow. While contact-mediated inhibition of cell proliferation is an important mechanism for controlling tissue homeostasis and size of solid organs, this phenomenon may have a lesser impact on cells in a liquid tissue. This may explain why YAP1 causes cell proliferation and overgrowth in solid tissues but not in the hematopoietic system.

In mammalian cells one of the most well characterized regulators upstream of the Hippo pathway is Neurofibromatosis 2/Merlin (Nf2/Mer). Nf2/Mer is required for contact inhibition in mammalian cells and restricts Yap1 activity in the liver [23]. Nf2 depletion in the liver causes a similar overgrowth phenotype as seen in livers of Mst1/Mst2 knockout mice or mice with ectopic expression of YAP1 [24]. By contrast, Nf2 depletion in hematopoietic cells has no cell autonomous effects on HSCs or progenitor cells, which is consistent with our observations of YAP1 overexpression in hematopoiesis [25]. However, Nf2 deficiency has profound consequences on several non-hematopoietic components of the bone marrow microenvironment, which is manifested by dysregulation of stroma cells, endothelium and bone, leading to secondary effects on localization and function of HSCs [25]. It is an interesting possibility that Nf2/Mer functions through Hippo signaling in this context, and it would therefore be of considerable interest to explore the role of YAP1 within the non-hematopoietic cells of the bone marrow microenvironment.

Although it is clear that increased YAP1 activity does not influence HSCs during conditions of steady state and regeneration, we cannot exclude changes in the HSC compartment that would have consequences under conditions of severe hematopoietic stress. When analyzing the expression of potential target genes in YAP^mut^ induced LSK cells we noted a down modulation of the cell cycle inhibitor p21 (data not shown). HSCs from mice lacking p21 show essentially normal function, but following severe hematopoietic stress by multiple serial transplantations become gradually exhausted [26]. Whether YAP1 induction and decreased p21, in that context, would influence the HSC pool remains to be investigated.

While our findings indicate that YAP1 does not affect HSC function, it is possible that a downstream mediator of the Hippo pathway, other than Yap1, is active in hematopoietic cells. In *Drosophila*, the Yap1 homolog Yorkie is the only known effector in the Hippo pathway. In mammalian cells, however, the evolutionary divergent Yap1 paralog Taz (Wwtr1) has partially overlapping functions with Yap1, forming complexes with TEAD transcription factors and mediates cell proliferation [27]. It has, however, been demonstrated that Yap and Taz has several distinct functions that are dependent on species and tissue type [28], [29], [30]. Consequently, it would be interesting to investigate whether Taz has an active role in regulation of hematopoeisis and HSCs. Indeed, our preliminary observations suggest that Taz is expressed at relatively high levels within the LT-HSC compartment but not in progenitors and differentiated hematopoietic cells (Jansson and Larsson unpublished observation).

In summary, our findings conclusively show that counteracting Hippo signaling by enforced YAP1 expression does not alter in vivo hematopoiesis or HSC function. More work will be required to elucidate whether other aspects of Hippo signaling, directly or indirectly, may influence hematopoietic cells, either through the Yap paralog Taz, or through actions on neighboring non-hematopoietic cells in the bone marrow microenvironment.

## Supporting Information

Figure S1 (**A**) Chromatogram from sequencing of pBS31 targeting vector with the mutated version of YAP1 (YAP^mut^). (**B**) Featured are the three bases making up the Serine 127 to Alanine conversion and the following two amino acids. (**C**) Intracellular flow cytometric analysis demonstrating YAP1 overexpression in ES cells with or without doxycycline and control FMO (Fluorescence minus one) with secondary anti-rabbit DyLight conjugated antibody.
(TIF)

Figure S2 **Wildtype and mutant YAP1 constructs show the expected phenotypes when induced in non-hematopoietic tissues.** ES cells with inducible wildtype YAP1 were injected into W41 blastocysts and YAP1 chimeric mice (n = 3) and wildtype control mice (n = 2) were administered doxycycline during 46 days before they were sacrificed and the liver appearance (**A**) and weight (**B**) examined. Upon doxycycline induction YAP1 chimeric mice developed hepatomegaly in accordance with known YAP functions in the liver (**C**) Germline YAP^mut^ mice with generalized YAP overexpression were induced with doxycycline in the drinking water. The graph shows percent survival for induced YAP^mut^ mice (n = 5) and littermate controls (n = 10). (**D**) Germline YAP^mut^ mice were induced with doxycycline for 4 days and then sacrificed. Total liver cells were used for RNA extraction, reverse transcription and RT qPCR. The expression of YAP1 and the YAP1 response gene Birc5 was measured. Error bars represent SEM for n = 2.
(TIF)

## References

[1] Justice RW, Zilian O, Woods DF, Noll M, Bryant PJ (1995). The Drosophila tumor suppressor gene warts encodes a homolog of human myotonic dystrophy kinase and is required for the control of cell shape and proliferation.. Genes Dev.

[2] Xu T, Wang W, Zhang S, Stewart RA, Yu W (1995). Identifying tumor suppressors in genetic mosaics: the Drosophila lats gene encodes a putative protein kinase.. Development.

[3] Huang J, Wu S, Barrera J, Matthews K, Pan D (2005). The Hippo signaling pathway coordinately regulates cell proliferation and apoptosis by inactivating Yorkie, the Drosophila Homolog of YAP.. Cell.

[4] Dong J, Feldmann G, Huang J, Wu S, Zhang N (2007). Elucidation of a universal size-control mechanism in Drosophila and mammals.. Cell.

[5] Hao Y, Chun A, Cheung K, Rashidi B, Yang X (2008). Tumor suppressor LATS1 is a negative regulator of oncogene YAP.. J Biol Chem.

[6] Zhang N, Bai H, David KK, Dong J, Zheng Y (2010). The Merlin/NF2 tumor suppressor functions through the YAP oncoprotein to regulate tissue homeostasis in mammals.. Dev Cell.

[7] Chan EH, Nousiainen M, Chalamalasetty RB, Schafer A, Nigg EA (2005). The Ste20-like kinase Mst2 activates the human large tumor suppressor kinase Lats1.. Oncogene.

[8] Hergovich A, Schmitz D, Hemmings BA (2006). The human tumour suppressor LATS1 is activated by human MOB1 at the membrane.. Biochem Biophys Res Commun.

[9] Webb C, Upadhyay A, Giuntini F, Eggleston I, Furutani-Seiki M (2011). Structural Features and Ligand Binding Properties of Tandem WW Domains from YAP and TAZ, Nuclear Effectors of the Hippo Pathway.. Biochemistry.

[10] Wang K, Degerny C, Xu M, Yang XJ (2009). YAP, TAZ, and Yorkie: a conserved family of signal-responsive transcriptional coregulators in animal development and human disease.. Biochem Cell Biol.

[11] Yagi R, Chen LF, Shigesada K, Murakami Y, Ito Y (1999). A WW domain-containing yes-associated protein (YAP) is a novel transcriptional co-activator.. EMBO J.

[12] Zhao B, Ye X, Yu J, Li L, Li W (2008). TEAD mediates YAP-dependent gene induction and growth control.. Genes Dev.

[13] Camargo FD, Gokhale S, Johnnidis JB, Fu D, Bell GW (2007). YAP1 increases organ size and expands undifferentiated progenitor cells.. Curr Biol.

[14] Ramalho-Santos M, Yoon S, Matsuzaki Y, Mulligan RC, Melton DA (2002). “Stemness”: transcriptional profiling of embryonic and adult stem cells.. Science.

[15] Cao X, Pfaff SL, Gage FH (2008). YAP regulates neural progenitor cell number via the TEA domain transcription factor.. Genes Dev.

[16] Alarcon C, Zaromytidou AI, Xi Q, Gao S, Yu J (2009). Nuclear CDKs drive Smad transcriptional activation and turnover in BMP and TGF-beta pathways.. Cell.

[17] Lian I, Kim J, Okazawa H, Zhao J, Zhao B (2010). The role of YAP transcription coactivator in regulating stem cell self-renewal and differentiation.. Genes Dev.

[18] Beard C, Hochedlinger K, Plath K, Wutz A, Jaenisch R (2006). Efficient method to generate single-copy transgenic mice by site-specific integration in embryonic stem cells.. Genesis.

[19] Ema H, Nakauchi H (2000). Expansion of hematopoietic stem cells in the developing liver of a mouse embryo.. Blood.

[20] Andrews NC, Faller DV (1991). A rapid micropreparation technique for extraction of DNA-binding proteins from limiting numbers of mammalian cells.. Nucleic acids research.

[21] Jansson L, Larsson J (2010). W41/W41 blastocyst complementation: a system for genetic modeling of hematopoiesis.. Blood.

[22] Oka T, Mazack V, Sudol M (2008). Mst2 and Lats kinases regulate apoptotic function of Yes kinase-associated protein (YAP).. J Biol Chem.

[23] Zhao B, Wei X, Li W, Udan RS, Yang Q (2007). Inactivation of YAP oncoprotein by the Hippo pathway is involved in cell contact inhibition and tissue growth control.. Genes Dev.

[24] Zhou D, Conrad C, Xia F, Park JS, Payer B (2009). Mst1 and Mst2 maintain hepatocyte quiescence and suppress hepatocellular carcinoma development through inactivation of the Yap1 oncogene.. Cancer Cell.

[25] Larsson J, Ohishi M, Garrison B, Aspling M, Janzen V (2008). Nf2/merlin regulates hematopoietic stem cell behavior by altering microenvironmental architecture.. Cell Stem Cell.

[26] Cheng T, Rodrigues N, Shen H, Yang Y, Dombkowski D (2000). Hematopoietic stem cell quiescence maintained by p21cip1/waf1.. Science.

[27] Zhang H, Liu CY, Zha ZY, Zhao B, Yao J (2009). TEAD transcription factors mediate the function of TAZ in cell growth and epithelial-mesenchymal transition.. J Biol Chem.

[28] Varelas X, Sakuma R, Samavarchi-Tehrani P, Peerani R, Rao BM (2008). TAZ controls Smad nucleocytoplasmic shuttling and regulates human embryonic stem-cell self-renewal.. Nat Cell Biol.

[29] Nishioka N, Inoue K, Adachi K, Kiyonari H, Ota M (2009). The Hippo signaling pathway components Lats and Yap pattern Tead4 activity to distinguish mouse trophectoderm from inner cell mass.. Dev Cell.

[30] Van Hateren NJ, Das RM, Hautbergue GM, Borycki AG, Placzek M (2011). FatJ acts via the Hippo mediator Yap1 to restrict the size of neural progenitor cell pools.. Development.

