# Supplementary figures and images for: Normal Hematopoietic Stem Cell Function in Mice with Enforced Expression of the Hippo Signaling Effector YAP1
Source: PLoS One. 2012 Feb 21;7(2):e32013. doi: 10.1371/journal.pone.0032013 (PMC3283704; doi:10.1371/journal.pone.0032013)

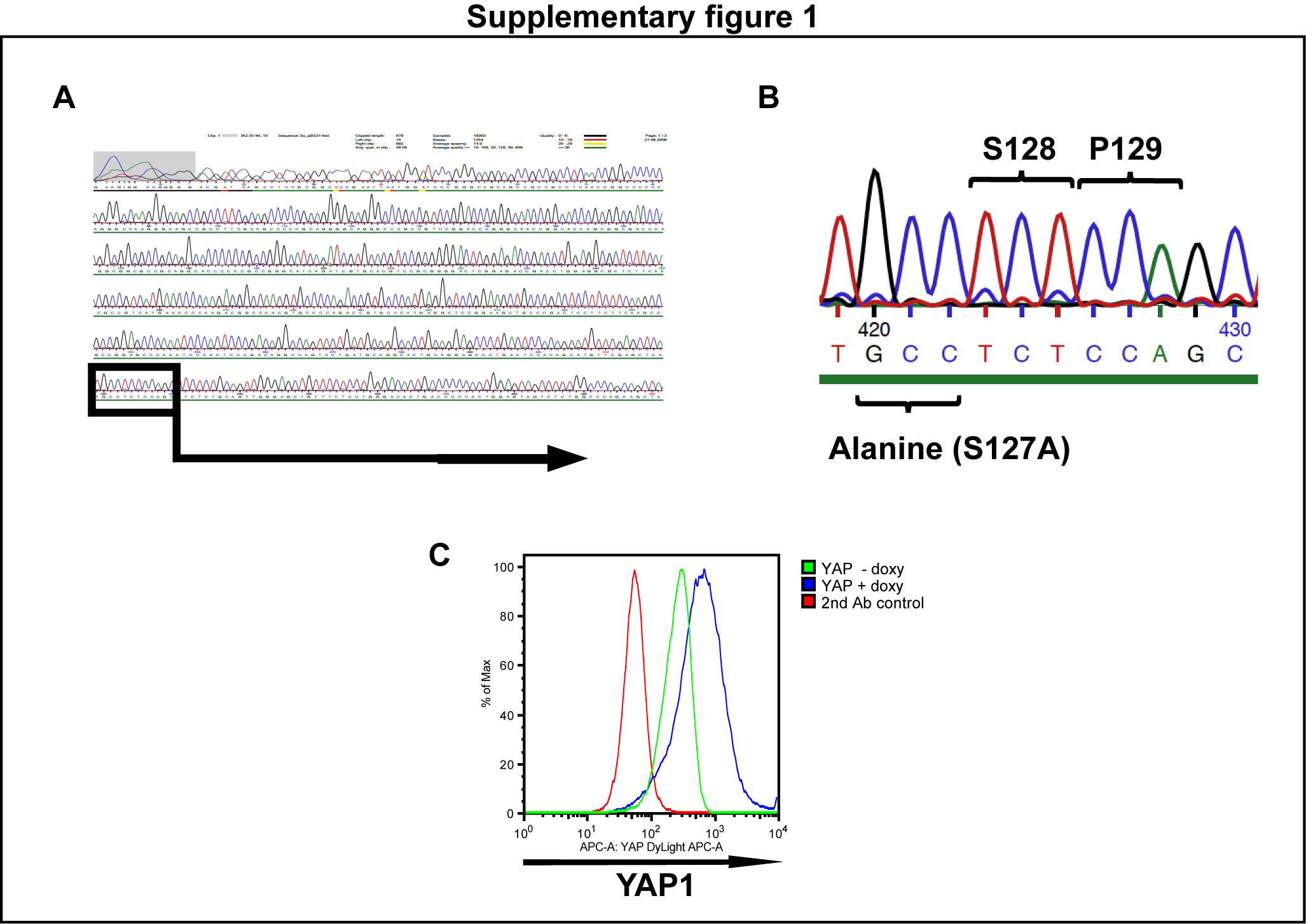

Supplement: Figure S1 — (A) Chromatogram from sequencing of pBS31 targeting vector with the mutated version of YAP1 (YAPmut). (B) Featured are the three bases making up the Serine 127 to Alanine conversion and the following two amino acids. (C) Intracellular flow cytometric analysis demonstrating YAP1 overexpression in ES cells with or without doxycycline and control FMO (Fluorescence minus one) with secondary anti-rabbit DyLight conjugated antibody. (TIF) [file pone.0032013.s001.tif]

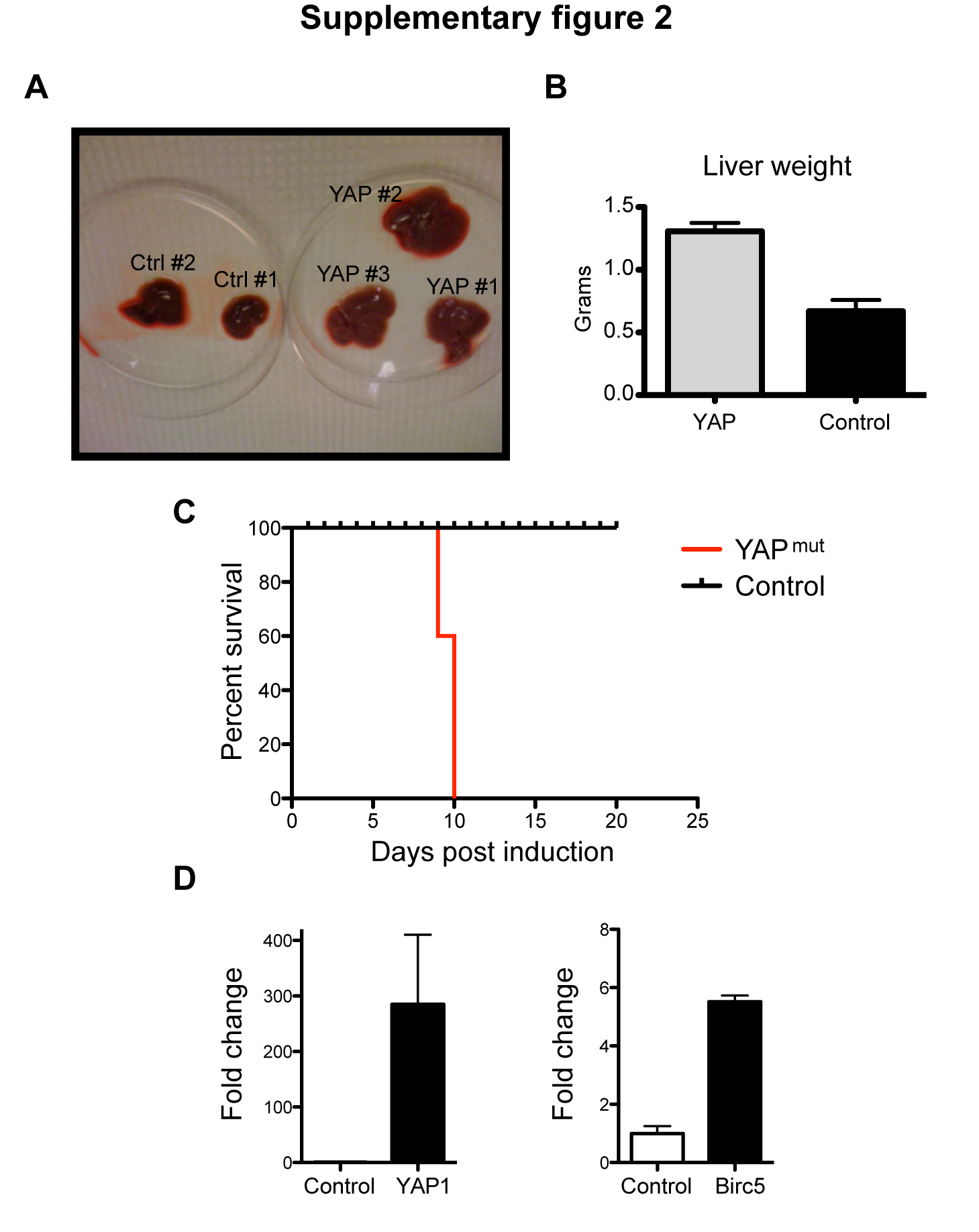

Supplement: Figure S2 — Wildtype and mutant YAP1 constructs show the expected phenotypes when induced in non-hematopoietic tissues. ES cells with inducible wildtype YAP1 were injected into W41 blastocysts and YAP1 chimeric mice (n = 3) and wildtype control mice (n = 2) were administered doxycycline during 46 days before they were sacrificed and the liver appearance (A) and weight (B) examined. Upon doxycycline induction YAP1 chimeric mice developed hepatomegaly in accordance with known YAP functions in the liver (C) Germline YAPmut mice with generalized YAP overexpression were induced with doxycycline in the drinking water. The graph shows percent survival for induced YAPmut mice (n = 5) and littermate controls (n = 10). (D) Germline YAPmut mice were induced with doxycycline for 4 days and then sacrificed. Total liver cells were used for RNA extraction, reverse transcription and RT qPCR. The expression of YAP1 and the YAP1 response gene Birc5 was measured. Error bars represent SEM for n = 2. (TIF) [file pone.0032013.s002.tif]
